# Does emotional intelligence mediates workplace stress, mindfulness, person-job-fit, and mental health among bank employees

**DOI:** 10.3389/fpsyg.2026.1896426

**Published:** 2026-08-26

**Authors:** Obinna Osita Ike, Ebele Evelyn Nnadozie, Elisha John Igwe

**Affiliations:** 1 Department of Psychology, University of Nigeria, Nsukka, Nsukka, Nigeria; 2 Department of Psychology, Alex Ekwueme Federal University Ndufu-Alike, Ikwo, Nigeria

**Keywords:** banking sector, emotional intelligence, mental health, mindfulness, person–job fit, workplace stress

## Abstract

**Introduction:**

Mental health challenges among bank employees represent a growing public health concern with substantial individual and organizational consequences. Guided by the Job Demands–Resources (JD-R) model and Emotion Regulation Theory, this study examined the mediating role of emotional intelligence (EI) in the relationships between workplace stress, trait mindfulness, and person–job fit (PJF) on employee mental health in the banking sector.

**Methods:**

Using a two-wave, time-lagged survey design, 412 bank employees (males = 159, 38.6%; females = 253, 61.4%; age range: 25–55 years; M = 35.64, SD = 7.30) were recruited from commercial banks in Southeast Nigeria via multi-stage sampling. Participants completed validated measures of workplace stress, mindfulness, person–job fit, emotional intelligence, and mental well-being. Data were analyzed using Hayes’ PROCESS macro (Model 4).

**Results:**

Results indicated that mindfulness was negatively associated with mental health, whereas EI and PJF demonstrated significant positive associations. Workplace stress showed no significant direct relationship with mental health. With respect to direct pathways, mindfulness negatively predicted EI, while workplace stress demonstrated a positive association with EI; PJF showed no significant direct effect on EI. Notably, EI significantly mediated the mindfulness–mental health pathway, partially reducing the adverse direct effect of mindfulness on mental health — a finding that challenges the prevailing assumption that mindfulness is uniformly beneficial in occupational contexts. EI did not mediate the PJF–mental health or workplace stress–mental health pathways.

**Discussion:**

These findings suggest that emotional intelligence (EI) serves as a psychological mechanism connecting mindfulness to employee mental health. This underscores the intricate and context-dependent nature of mindfulness, particularly in high-demand work environments such as the banking sector.

## Introduction

Employee mental health has emerged as a pressing global concern, recognized as fundamental to both individual flourishing and organizational sustainability (Ballard et al., 2025; World Health Organization, 2022). Mental health is broadly understood as a state of emotional, psychological, and social well-being that shapes how individuals think, feel, make decisions, and relate to others in the workplace (Keyes, 2002). In increasingly volatile and performance driven work environments, adverse mental health outcomes—including anxiety, burnout, and emotional exhaustion—have become widespread, imposing significant costs on both individuals and organizations (American Psychological Association, 2023; Deady et al., 2022).

Globally, employees in the banking sector encounter mounting pressures stemming from digital transformation, heightened customer expectations, regulatory requirements, and job insecurity, all of which contribute to increased workplace stress and compromised mental health (Ribeiro et al., 2024; Zhang and Lee, 2023). The rapid expansion of digital banking has significantly altered job demands, requiring continuous technological adaptation, increased multitasking, and higher service responsiveness, thereby intensifying work-related strain (Alam and Hassan, 2024). Regulatory compliance demands, cybersecurity concerns, and organizational restructuring associated with financial digitalization further compound occupational stress (Ribeiro et al., 2024) and psychological fatigue (Chen and Cao, 2023). These cumulative stressors have been linked to burnout, reduced job satisfaction, and deteriorating mental health outcomes across workforces worldwide (World Health Organization, 2023).

In Nigeria, these challenges are often compounded by a socio-economic context characterized by economic instability, inflationary pressures, workforce restructuring, and rigorous performance monitoring. Recent evidence indicates that Nigeria’s macroeconomic volatility—including high inflation, currency depreciation, and fiscal adjustments—has intensified pressures across the financial sector (African Development Bank, 2024; World Bank, 2024). Employees are frequently tasked with achieving demanding sales targets under conditions of limited staffing, long working hours, and increasing workload demands, often with inadequate psychosocial support, which significantly heightens job stress (Ajayi, 2018; Iyiegbuniwe and Imoni, 2020; Okon et al., 2021). These circumstances exacerbate vulnerability to chronic stress, emotional exhaustion, and deteriorating mental health outcomes, including burnout and reduced job satisfaction among banking employees in Nigeria (Nwokolo, 2025; Oseremen et al., 2022). Moreover, organizational mental health policies remain underdeveloped in many under developed economies such as in Nigerian workplaces, compelling employees to rely predominantly on individual coping mechanisms rather than structured institutional support (Adeniji et al., 2023; Yunusa et al., 2024). Commercial banking combines intense performance targets, regulatory scrutiny, role ambiguity, and high emotional labor demands, making bank employees particularly susceptible to occupational stress and poor psychological well-being (Undie et al., 2024). In Nigeria specifically, empirical evidence documents a strong association between workplace stress and adverse mental health outcomes among bank employees, with studies reporting high rates of emotional exhaustion, burnout, and anxiety (Ebhote et al., 2022; Fasoranti et al., 2021). Understanding the determinants of employee mental health in this sector is therefore both scientifically important and practically urgent.

A substantial body of literature identifies workplace stress as one of the most significant association of employee mental health. Drawing on the Job Demands–Resources (JD-R) model (Bakker and Demerouti, 2017; Demerouti and Bakker, 2023), workplace stress represents a core job demand that depletes employees’ psychological resources, thereby increasing the risk of burnout, anxiety, and diminished well-being. However, the extent to which stress impacts mental health is not uniform; it is modulated by personal and contextual resources that shape how demands are perceived and managed (Schaufeli and Taris, 2014). Among these resources, mindfulness has attracted growing scholarly attention for its role in buffering occupational stress and promoting psychological well-being. Defined as present-moment, non-judgmental awareness (Kabat-Zinn, 2003), mindfulness facilitates adaptive emotional regulation, cognitive flexibility, and reduced rumination (Atta et al., 2024; Hofmann et al., 2010). Empirical work consistently demonstrates that mindfulness is negatively related to stress and burnout and positively associated with mental health across diverse occupational contexts (Wang et al., 2023; Dhaka et al., 2024; Long et al., 2025). Mindfulness-based interventions have similarly yielded significant reductions in stress and improvements in job satisfaction (Lomas et al., 2017; Blodgett et al., 2022).

Furthermore, alongside individual psychological resources, organizational factors play an equally critical role in shaping employee mental health. Person–job fit (PJF)—defined as the alignment between an individual’s skills, abilities, and values and the demands and characteristics of their job (Edwards, 1991)—is a key organizational resource associated with job satisfaction, engagement, and psychological well-being (Kristof-Brown et al., 2005). Conversely, person–job misfit is associated with role strain, frustration, and elevated stress, all of which undermine mental health (Zeng and Hu, 2024; Brandstätter et al., 2016).

Despite established direct effects of mindfulness, PJF, and workplace stress on mental health, the psychological processes linking these variables remain insufficiently understood. Emotional intelligence (EI)—defined as the capacity to perceive, understand, regulate, and utilize emotions effectively (Mayer and Salovey, 1997)—has been identified as a mediating link (Galea et al., 2025; Li et al., 2023). Studies show that individuals with higher EI are better positioned to reappraise stressors, manage negative affect, and sustain positive mental health (Han et al., 2025). Empirical evidence also suggests that mindfulness enhances EI by increasing emotional awareness and promoting deliberate rather than reactive emotional responses (Brun et al., 2023; Schutte et al., 2021), while sustained workplace stress may impair EI by overwhelming regulatory resources (He et al., 2024). Existing research has largely examined these constructs in isolation, with limited integration of mindfulness, PJF, workplace stress, and EI within a single, theoretically coherent framework. The mediating role of EI in linking these antecedents to employee mental health has not been simultaneously tested, leaving important gaps in understanding the processes through which individual and organizational factors jointly influence psychological well-being.

The present study addresses these gaps by proposing and testing an integrative mediation model that specifies EI as the psychological pathway through which resources (mindfulness, PJF) and demands (workplace stress) are translated into mental health outcomes. Situated within a neglected under explored context, the study draws on the JD-R model (Bakker and Demerouti, 2017; Demerouti and Bakker, 2023) and Emotion Regulation Theory (Gross, 2015) to provide a theoretically grounded examination of these pathways. By doing so, the study makes both theoretical and practical contributions to work and organizational psychology literature, responding to calls for more diverse and contextualized research in under developed contexts such as sub-Saharan African settings (Alarcon-Espinoza et al., 2022), and informing evidence-based organizational interventions aimed at enhancing employee mental health.

## Theoretical framework and hypotheses development

The present study is grounded in two complementary theoretical frameworks: the Job Demands–Resources (JD-R) model (Bakker and Demerouti, 2017) and Emotion Regulation Theory (Gross, 2015, 2024). The JD-R model provides the primary structural framework by positing that employee mental health is shaped by two broad classes of work characteristics: job demands—the physical, cognitive, or emotional requirements that impose psychological costs—and job resources—the physical, social, or organizational features that reduce demands, facilitate goal achievement, and support personal growth. The model further distinguishes between job resources (e.g., autonomy, supervisor support) and personal resources (e.g., self efficacy, resilience), both of which moderate the demand–strain relationship (Schaufeli and Taris, 2014; Demerouti and Bakker, 2023). Within this framework, workplace stress is conceptualized as a central job demand. When employees perceive that job requirements exceed their coping capacities, psychological strain results, manifesting as anxiety, emotional exhaustion, and diminished well-being (Bakker and Demerouti, 2017). Empirical support for the direct negative effect of workplace stress on mental health is robust and cross-cultural (Deady et al., 2022; Schaufeli and Taris, 2014). Mindfulness and PJF, by contrast, are conceptualized as resources within the JD-R model. Mindfulness functions as a personal resource that enhances attentional regulation and reduces maladaptive stress reactivity (Dhaka et al., 2024; Long et al., 2025), while PJF acts as a job resource that promotes feelings of competence, role clarity, and reduced strain (Kristof-Brown et al., 2005; Zeng and Hu, 2024). The JD-R model thus predicts positive direct effects of mindfulness and PJF on mental health, and a negative direct effect of workplace stress. Notably, the JD-R model also accommodates personal resources such as EI, which the present study proposes as a mediator linking mindfulness, person–job fit (PJF), and workplace stress to mental health. Employees with high EI are better equipped to leverage available resources and buffer against demands, facilitating mental health and psychological well-being (Galea et al., 2025; Li et al., 2023).

Emotion Regulation Theory (Gross, 2015, 2024) provides further theoretical support. Emotion regulation refers to the processes by which individuals influence which emotions they have, when they have them, and how they experience and express those emotions (Gross, 2015). Maladaptive emotion regulation—characterized by suppression, rumination, and emotional reactivity—is strongly associated with psychological distress, anxiety, and poor mental health (Christopher and Facal, 2023; Valenzuela et al., 2025). Adaptive regulation strategies, including cognitive reappraisal and acceptance, are associated with positive mental health outcomes (Giancola et al., 2024; Lewczuk et al., 2022). EI is positioned within Emotion Regulation Theory as a core regulatory capability that shapes how individuals appraise, experience, and manage emotional responses to organizational demands. Individuals with higher EI engage in more adaptive regulatory strategies, reframe stressful situations constructively, and maintain psychological stability under adversity (Galea et al., 2025; He et al., 2024). Mindfulness is known to enhance EI by reducing automatic emotional reactivity and increasing emotional clarity (Brun et al., 2023; Jiménez-Picón et al., 2021; Schutte et al., 2021). Conversely, sustained workplace stress depletes regulatory resources and undermines EI functioning (He et al., 2024; Lewczuk et al., 2022). By integrating the JD-R model with.

Emotion Regulation Theory, the present study constructs a comprehensive framework in which EI mediates the transformation of demands and resources into mental health outcomes.

## Literature review

### Workplace stress and mental health

Workplace stress—defined as the physiological and psychological strain that arises when perceived job demands exceed an individual’s coping resources—is one of the most extensively documented predictors of employee mental health (Bakker and Demerouti, 2017; Deady et al., 2022). Research consistently demonstrates strong associations between occupational stressors and adverse outcomes including depression, anxiety, burnout, and emotional exhaustion across a wide range of industries and cultural contexts (Schaufeli and Taris, 2014; World Health Organization, 2022). In the banking sector, workplace stress is a particularly salient concern. Banking employees routinely face excessive workloads, aggressive performance targets, role ambiguity, and high emotional labor demands, all of which create conditions conducive to psychological strain (Undie et al., 2024). Studies conducted in African context, document significant links between occupational stress and mental health deterioration, including depression and emotional exhaustion (Ebhote et al., 2022; Fasoranti et al., 2021) among these category of people. For instance, in Nigeria, research indicates that employee turnover among banks rose sharply between 2023 and 2024, driven in large part by stress-related psychological attrition (Bernard, 2025). Beyond documenting direct effects, recent scholarship has sought to identify the pathway through which workplace stress association with mental health. The depletion of personal regulatory resources—including emotional regulation capacity—is increasingly recognized as a critical pathway linking sustained stress to psychological impairment (Gross, 2024; Lewczuk et al., 2022). Under conditions of chronic occupational stress, individuals experience cognitive overload and emotional fatigue, diminishing their capacity for adaptive coping (He et al., 2024). This underscores the importance of EI as a mediating resource that determines whether stress link to mental health deterioration or is managed effectively.

### Mindfulness and mental health

Mindfulness—broadly defined as present-moment awareness adopted with an attitude of openness and non-judgment (Kabat-Zinn, 2003)—has emerged as a robust predictor of mental health in occupational contexts. A large-scale meta-analysis by Lomas et al. (2017), reviewing 153 studies involving over 12,000 participants, found that mindfulness-based interventions consistently reduced psychological stress and improved job satisfaction. More recent systematic reviews and meta-analyses confirm these effects, with mindfulness-based interventions associated with significant reductions in burnout, anxiety, and depression among employees (Selič-Zupančič et al., 2023; Wang et al., 2023). In the existing literature, mindfulness is conceptualized from three interrelated perspectives: as a stable dispositional characteristic known as trait mindfulness, as a transient state of present-moment awareness referred to as state mindfulness, and as a trainable practice developed through mindfulness based interventions (Good et al., 2016; Brown and Ryan, 2003; Tanay and Bernstein, 2013).

At the dispositional level, trait mindfulness refers to an individual’s stable tendency to sustain present-moment awareness in a non-judgmental and nonreactive manner, representing a relatively enduring individual difference variable (Brown and Ryan, 2003). At the situational level, state mindfulness denotes a transient, context-dependent experience of present-focused awareness that fluctuates within individuals in response to circumstance or intentional practice (Tanay and Bernstein, 2013). A third perspective conceptualizes mindfulness as a deliberate, skill-based practice — encompassing structured techniques such as meditation, breath-focused attention, and body scanning — that forms the foundation of evidence-based programs including Mindfulness-Based Stress Reduction (MBSR) and Mindfulness-Based Cognitive Therapy (MBCT; Kabat-Zinn, 1990; Segal et al., 2002). These perspectives are theoretically distinct yet functionally interrelated. Empirical evidence consistently associates higher levels of these mindfulness states with superior psychological functioning, including reduced anxiety and depressive symptomatology, elevated life satisfaction, and accelerated affective recovery following stressor exposure (Alvarado-García et al., 2025; Chua et al., 2025; Fortes et al., 2025; Pan and Yi, 2024). Mindfulness facilitates mental health through several interrelated mechanisms. It enables individuals to disengage from ruminative thought patterns and respond to stressors adaptively rather than automatically (Atta et al., 2024; Giancola et al., 2024). Research demonstrates that mindful individuals are more likely to employ cognitive reappraisal and less likely to rely on suppression or avoidance as regulatory strategies (Giancola et al., 2024; Hofmann et al., 2010). In organizational contexts, mindfulness has been shown to reduce emotional exhaustion, enhance resilience, and buffer the negative effects of workplace incivility and interpersonal conflict (Dhaka et al., 2024; Robinson and Krishnakumar, 2022). The relationship between mindfulness and EI is particularly well established. Cross-sectional and longitudinal studies consistently demonstrate that higher mindfulness is associated with greater.

EI, as mindful awareness strengthens the capacity to recognize, understand, and regulate emotional experiences (Jiménez-Picón et al., 2021; Schutte et al., 2021). In a longitudinal mixed-methods study, Han et al. (2025) found that mindfulness practice was negatively associated with emotional exhaustion and that EI served as a key protective factor through which the relationship was mediated. Brun et al. (2023) and Hsu et al. (2024) further confirm that mindfulness enhance EI-related competencies among professionals in high-demand roles.

### Person–job fit and mental health

Person–job fit (PJF) refers to the compatibility between an individual’s knowledge, skills, abilities, and values and the requirements and characteristics of their job role (Edwards, 1991; Kristof-Brown et al., 2005). Strong PJF is associated with a sense of competence, autonomy, and meaningfulness that directly promotes mental health (Brandstätter et al., 2016). Empirically, high PJF associates with greater job satisfaction, engagement, and positive affect, while poor PJF is associated with frustration, role strain, and emotional exhaustion (Zeng and Hu, 2024). In the banking context, the risk of person–job misfit is elevated given the prevalence of role ambiguity, excessive multitasking, and rapid organizational restructuring (Ebhote et al., 2022). Employees frequently find themselves assigned responsibilities that fall outside their core competencies or values, increasing role-related stress and psychological strain. Empirical research in the neglected under explored context supports PJF as a significant predictor of reduced stress and enhanced well-being in professional service occupations (Fasoranti et al., 2021). The relationship between PJF and EI has received less empirical attention but is theoretically coherent within both the JD-R model and Emotion Regulation Theory. Employees who perceive strong alignment between their abilities and job demands are more likely to experience positive emotional states—including confidence, flow, and intrinsic motivation—that support the development and maintenance of EI competencies (Gross, 2024; Zeng and Hu, 2024). However, chronic role-ability mismatch may generate frustration and emotional strain that erodes regulatory capacity. These theoretical arguments are noted, though empirical evidence for this pathway remains sparse, rendering its test a key contribution of the present study.

### Emotional intelligence as a mediator

Emotional intelligence—conceptualized by Mayer and Salovey (1997) as the ability to perceive, use, understand, and manage emotions—is consistently identified as a critical determinant of employee mental health in occupational contexts. Individuals with high EI engage more effectively in adaptive coping, maintain positive interpersonal relationships, and demonstrate greater resilience under stressful conditions (Galea et al., 2025; He et al., 2024). Meta-analytic evidence confirms EI as a significant predictor of reduced burnout, lower anxiety, and enhanced well-being across professional contexts (Li et al., 2023). The mediating role of EI in connecting organizational antecedents to mental health outcomes is supported by a growing body of research. For instance, Li et al. (2023) found that EI mediated the relationship between mindfulness and occupational burnout among intensive care nurses, suggesting that EI captures the mechanism through which mindfulness translates into reduced psychological strain. Long et al. (2025) similarly demonstrated that EI was a key pathway through which mindfulness influenced work performance and well-being. This body of evidence provides strong empirical support for the proposed mediation of EI in the mindfulness–mental health relationship. With respect to workplace stress, research demonstrates that high stress levels impair cognitive functioning and emotional regulation capacity, thereby reducing EI-related competencies (He et al., 2024; Lewczuk et al., 2022). Employees under chronic stress experience reduced self-awareness, diminished empathic accuracy, and impaired impulse control—all core components of EI (Galea et al., 2025). Whether EI mediates the stress–mental health relationship versus serving as a moderator or being independently affected by stress requires further empirical clarification. In summary, converging theoretical and empirical evidence positions EI as a plausible pathway in the relationships among workplace stress, mindfulness, PJF and employee mental health.

## The present study

The present study develops and tests an integrative mediation model of employee mental health among bank employees, particularly in an under explored context. Responding to increasing scholarly demands for theoretically grounded, process-oriented models of occupational wellbeing (Ballard et al., 2025; Demerouti and Bakker, 2023), the study simultaneously examines the roles of workplace stress, mindfulness and PJF on mental health, and tests whether these effects operate through EI as a mediating pathway. The banking sector, particularly in a neglected under explored African context provides an ecologically valid and theoretically pertinent setting for this investigation, as commercial banking combines structural features— performance pressure, regulatory demands, customer-facing emotional labor, role ambiguity, and rapid organizational change—that generate substantial occupational stress and place acute demands on employees’ emotional regulatory resources (Bernard, 2025; Undie et al., 2024).

Despite growing recognition of these individual relationships, a significant gap persists in understanding the combined, process-oriented dynamics of workplace stress, mindfulness, job-fit and EI as an integrated framework for mental health outcomes among banking professionals, particularly in sub-Saharan Africa. Few studies have explored the psychological processes through which individual and organizational factors collectively relates to employee mental health in the banking sector, particularly within non-Western contexts. Existing research has predominantly concentrated on isolated predictors of mental health and has largely relied on cross-sectional designs conducted in Western societies, thereby limiting temporal precision and causal inference (Giorgi et al., 2017). Recent reviews further indicate that significant gaps persist regarding the interaction of psychosocial, organizational, and personal factors affecting bankers’ mental health, especially in emerging economies and developing countries (Giorgi et al., 2017; Ndengu and Leka, 2022). Moreover, many studies in the banking sector continue to employ cross-sectional survey methods, which constrain the ability to establish directional and longitudinal relationships among study variables (Taris et al., 2021;

Giorgi et al., 2017). The aim of the present study is therefore to examine the mediating role of EI in the relationships among workplace stress, mindfulness, and PJF on mental health among bank employees in such neglected under explored context, using wave design. Aligning with prior theory and empirical evidence, the following hypotheses were proposed:

*H1*: Workplace stress negatively associated with employee mental health.

*H2*: Mindfulness positively associated with employee mental health.

*H3*: Person–job fit positively associated with employee mental health.

*H4*: Workplace stress negatively associated with emotional intelligence.

*H5*: Mindfulness positively associated with emotional intelligence.

*H6*: Person–job fit positively associated with emotional intelligence.

*H7*: Emotional intelligence positively associated with employee mental health.

*H8*: Emotional intelligence mediates the relationship between workplace stress and employee mental health.

*H9*: Emotional intelligence mediates the relationship between mindfulness and employee mental health.

*H10*: Emotional intelligence mediates the relationship between person–job fit and employee mental health.

**Figure 1:**
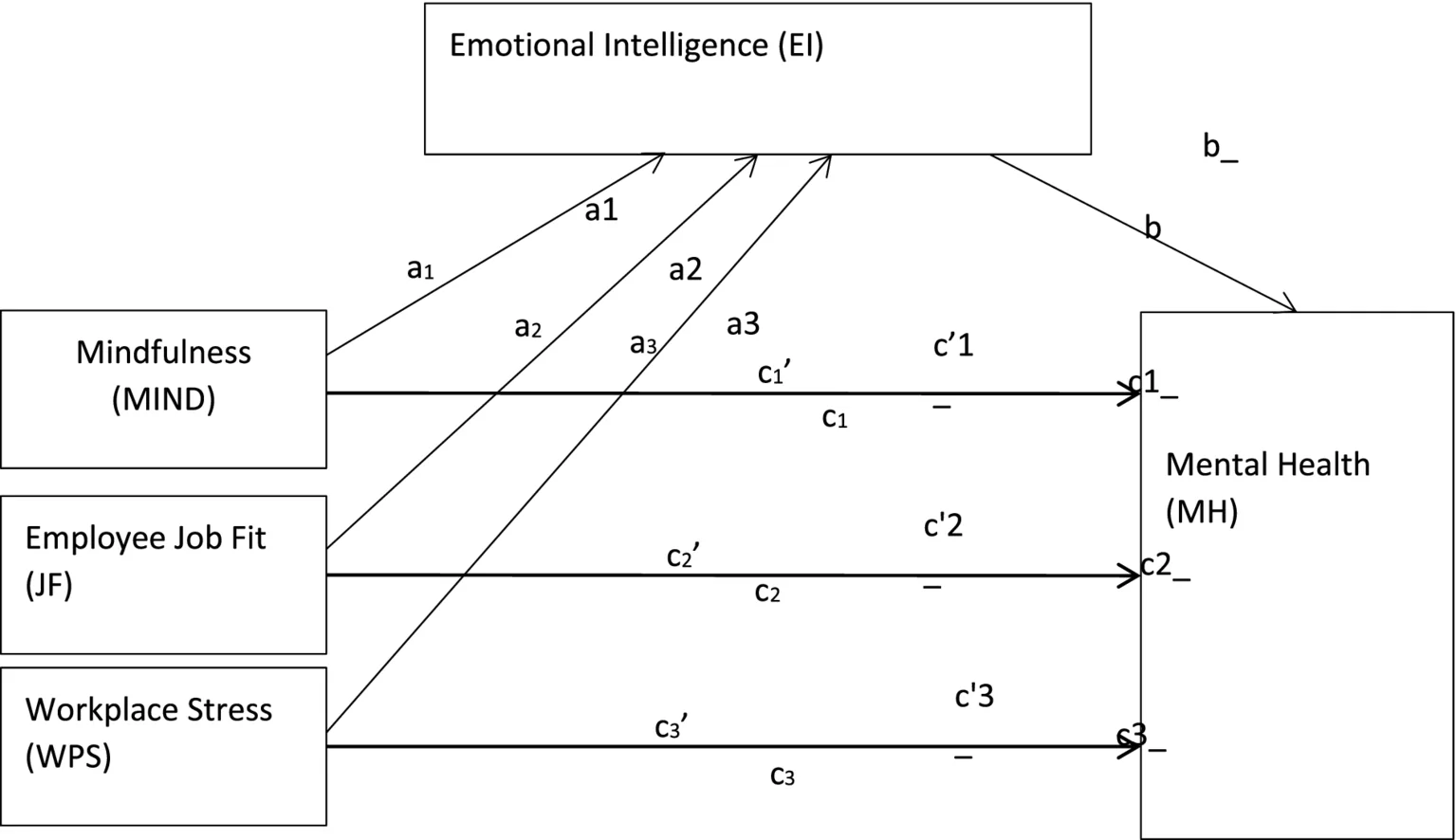
Hypothetical model for the mediating role of emotional intelligence in the relationships of mindfulness, employee job-fit, and workplace stress on mental health among bank employees.

Figure 1 presents the hypothetical structural model proposed in the present study, depicting the mediating role of emotional intelligence in the relationships between three independent variables — mindfulness, person- job fit, and workplace stress — and the outcome variable of mental health among bank employees. The model is grounded in a mediation framework in which emotional intelligence is positioned as an intervening mechanism through which the antecedent variables are linked to mental health outcomes. Specifically, the model specifies three direct pathways from the independent variables to emotional intelligence (paths a1, a2, and a3, representing the effects of mindfulness, employee job fit, and workplace stress, respectively), and a single direct pathway from emotional intelligence to mental health (path b). In addition, the model includes direct pathways from each independent variable to mental health (paths c1, c2, and c3), representing the unmediated effects, alongside the corresponding indirect pathways (c1’, c2’, and c3’), which capture the portion of each relationship through emotional intelligence. This model allows for the simultaneous examination of both direct and indirect effects, consistent with contemporary mediation analysis procedures. The model was tested with emotional intelligence hypothesized to partially or fully mediate the relationship of mindfulness, job fit, and workplace stress on mental health among the study sample.

## Methodology

### Participants and procedures

A total of 412 bank employees comprising 159 males (38.6%) and 253 females (61.4%), with an age range of 25–55 years (*M* = 35.64; SD = 7.3), participated in the study. Participants were selected through multi-stage sampling to ensure adequate representation of the target population from commercial banks in Southeast Nigeria. At the first stage, banks were purposively selected based on their operational license and widespread presence within the study area. In the second stage, branches of the selected banks were stratified by geographical location and randomly selected using simple random sampling. At the third stage, employees from the selected branches were conveniently sampled. Regarding demographics, 141 (34.2%) were single and 271 (65.8%) were married. For educational qualification, 210 (51.0%) held bachelor’s degrees, 11 (2.7%) held postgraduate degrees, and 191 (46.4%) held OND/HND qualifications.

Ethical approval for this study was obtained from the Institutional Ethical Research Committee (blinded for review), and formal permission was subsequently secured from the management of each participating bank prior to commencing data collection. All participants received written and verbal information describing the purpose of the study, the voluntary nature of their participation, the right to withdraw at any point without consequence, and the confidentiality and anonymity with which their responses would be handled. Informed consent was obtained from each participant before data collection began. Data were collected using a two-wave, time-lagged design with a four-week interval between assessments, consistent with recommendations for reducing common method bias in self-report research (Podsakoff et al., 2003). This design allowed the predictor and mediator variables to be assessed temporally prior to the outcome variable, thereby strengthening the directionality of inferences drawn from the mediation analyses. At Time 1 (T1), copies of the questionnaire assessing the independent variables (workplace stress, mindfulness and PJF), the mediating variable (EI), and relevant demographic characteristics were distributed to 440 bank employees across participating institutions. All 440 questionnaires were completed and returned, yielding a T1 response rate of 100%. Four weeks later, at Time 2 (T2), copies of the questionnaire assessing the dependent variable (mental health) were distributed to the same participants. To preserve anonymity while enabling accurate cross-wave matching of responses, participants had been asked at T1 to generate a unique self-selected identification code. Of the 440 participants approached at T2, 412 returned fully completed and matched questionnaires, yielding a T2 response rate of 93.6%. The remaining 28 participants did not complete the second wave, constituting attrition rate of 6.4%. Only participants with complete, matched responses across both waves were retained for analysis, resulting in an analytic sample of *N* = 412.

### Measures

#### Workplace stress

Workplace stress was assessed using the Job Stress Scale (JSS; Parker and DeCotiis, 1983), a validated 13-item self-report instrument comprising two subscales: time stress (8 items) capturing workload pressure and urgency, and anxiety (5 items) reflecting psychological tension from work demands. Items were rated on a four-point Likert scale (1 = strongly disagree to 4 = strongly agree), with higher scores indicating greater occupational stress. A composite total score was computed by summing responses across both subscales (Jamal, 2011; Karimi and Nouri, 2009). The JSS demonstrates robust psychometric properties across diverse occupational samples, with Cronbach’s alpha values ranging from 0.80 to 0.92.

The JSS exhibited excellent model fit indices, with χ^2^/df = 1.44, RMSEA = 0.041, SRMR = 0.032, CFI = 0.97, and TLI = 0.95, all of which indicate very good to excellent model fit. These values suggest that the measurement model fits the observed data exceptionally well. The construct also demonstrated acceptable internal consistency reliability (CR = 0.732), exceeding the minimum recommended threshold of 0.70. In addition, the AVE value (0.610) was well above the 0.50 criterion, indicating strong convergent validity and suggesting that the construct captures a substantial proportion of variance in its indicators. Overall, the JSS demonstrated excellent model fit and strong psychometric properties (see Table 1).

**Table 1:** Confirmatory factor analysis model fit indices, composite reliability, and average variance extracted for the study constructs.

Construct	χ^2^/df	RMSEA	SRMR	CFI	TLI	CR	AVE
Emotional intelligence	1.85	0.052	0.045	0.94	0.92	0.806	0.591
Mental Health	1.92	0.055	0.048	0.93	0.91	0.768	0.539
Job Stress	1.44	0.041	0.032	0.97	0.95	0.732	0.610
Mindfulness	1.61	0.047	0.038	0.95	0.94	0.712	0.587
Job Fit	1.73	0.050	0.041	0.92	0.90	0.848	0.532

χ^2^/df = normed chi-square (chi-square divided by degrees of freedom); RMSEA = Root Mean Square Error of Approximation; SRMR = Standardized Root Mean Square Residual; CFI = Comparative Fit Index; TLI = Tucker–Lewis Index; CR = Composite Reliability; AVE = Average Variance Extracted. Recommended thresholds for acceptable model fit are χ^2^/df < 3.00, RMSEA ≤ 0.08, SRMR ≤ 0.08, CFI ≥ 0.90, and TLI ≥ 0.90. Composite reliability values of 0.70 or higher indicate satisfactory internal consistency, while AVE values of 0.50 or higher indicate adequate convergent validity (Hair et al., 2019; Fornell and Larcker, 1981).

#### Mindfulness

Mindfulness was assessed using the 14-item short form of the Freiburg Mindfulness Inventory (FMI; Walach et al., 2006), a validated self-report measure capturing present-moment awareness, non-judgmental observation, and openness to experience. Unlike clinically focused mindfulness instruments, the FMI was designed for both meditating and general non-clinical samples, making it well-suited to occupational research contexts (Kohls et al., 2009). Items were rated on a four-point Likert scale (1 = rarely to 4 = almost always), with higher mean scores reflecting greater dispositional mindfulness. The FMI demonstrates strong psychometric properties, with Cronbach’s alpha values ranging from 0.83 to 0.91 across nonclinical and professional samples (Walach et al., 2006; Kohls et al., 2009; Sauer et al., 2013). The FMI showed good model fit, with χ^2^/df = 1.61, RMSEA = 0.047, SRMR = 0.038, CFI = 0.95, and TLI = 0.94, indicating that the hypothesized measurement model adequately represents the observed data. The composite reliability coefficient (CR = 0.712) met the minimum acceptable threshold of 0.70, demonstrating satisfactory internal consistency. Furthermore, the AVE value (0.587) exceeded the recommended cut-off of 0.50, confirming adequate convergent validity. These results indicate that the FMI possesses acceptable reliability and a well-fitting factor structure, making it suitable measure of mindfulness in this sample (see Table 1).

#### Person–job fit

Person–job fit was assessed using the five-item Perceived Ability– Job Fit Scale (PAJFS; Lauver and Kristof-Brown, 2001), a validated self-report instrument measuring employees’ subjective perceptions of congruence between their knowledge, skills, and abilities and their job demands. Items were rated on a five-point Likert scale (1 = strongly disagree to 5 = strongly agree), with higher mean scores indicating greater perceived ability– job fit. The PAJFS demonstrates strong psychometric properties, with Cronbach’s alpha values ranging from 0.87 to 0.93 across professional and service sector samples (Lauver and Kristof-Brown, 2001; Tak, 2011; Cable and DeRue, 2002). The PAJFS demonstrated acceptable model fit, with χ^2^/df = 1.73, RMSEA = 0.050, SRMR = 0.041, CFI = 0.92, and TLI = 0.90, all of which fall within acceptable thresholds for model adequacy. In terms of reliability, the construct showed strong internal consistency (CR = 0.848), exceeding the minimum recommended value of 0.70. Furthermore, the AVE value (0.532) surpassed the recommended cut-off of 0.50, indicating adequate convergent validity and suggesting that the construct explains a substantial proportion of variance in its indicators. In all, the PAJFS demonstrated a well-fitting measurement structure with satisfactory reliability and convergent validity (see Table 1).

#### Emotional intelligence

Emotional intelligence was assessed using the Brief Emotional Intelligence Scale (BEIS-10; Davies et al., 2010), a 10-item self-report instrument measuring EI across five subscales: appraisal of own emotions, appraisal of others’ emotions, regulation of own emotions, regulation of others’ emotions, and utilization of emotions. Items were rated on a five-point Likert scale (1 = strongly disagree to 5 = strongly agree), with higher scores reflecting greater EI. The BEIS-10 demonstrates acceptable to good psychometric properties, with Cronbach’s alpha values ranging from 0.75 to 0.84 across diverse professional samples and a confirmed five-factor structure (Davies et al., 2010; Sánchez-Álvarez et al., 2016). The Brief Emotional Intelligence Scale (BEIS-10) demonstrated acceptable psychometric properties based on the confirmatory factor analysis (CFA) results. The model exhibited good fit indices (χ^2^/df = 1.85, RMSEA = 0.052, SRMR = 0.045, CFI = 0.94, TLI = 0.92), all of which fall within recommended thresholds for acceptable model fit, indicating that the hypothesized factor structure adequately represents the observed data. In terms of reliability, the construct showed strong internal consistency (CR = 0.806), exceeding the minimum recommended value of 0.70. Furthermore, the AVE value (0.591) surpassed the recommended cut-off of 0.50, indicating adequate convergent validity and suggesting that the construct explains a substantial proportion of variance in its indicators. In all, the BEIS demonstrated a well-fitting measurement structure with satisfactory reliability and convergent validity (See Table 1).

#### Mental health

Mental health was assessed using the Warwick–Edinburgh Mental Well-being Scale (WEMWBS; Tennant et al., 2007), a 14-item self-report instrument measuring positive mental health across hedonic well-being (positive affect, vitality, and life satisfaction) and eudaimonic well-being (psychological functioning, personal growth, and sense of purpose). Participants rated each item over the preceding 2 weeks on a five-point Likert scale (1 = none of the time to 5 = all of the time), with total scores summed across all 14 items (range: 14–70), where higher scores indicate greater well-being. The WEMWBS demonstrates exceptional psychometric properties, with Cronbach’s alpha values of 0.89 to 0.91 and a confirmed unidimensional factor structure across diverse populations (Tennant et al., 2007; Perera et al., 2025). The WEMWBS demonstrated acceptable model fit, with χ^2^/df = 1.92, RMSEA = 0.055, SRMR = 0.048, CFI = 0.93, and TLI = 0.91, all of which meet established criteria for good model fit. These results indicate that the measurement model provides an adequate representation of the underlying construct structure. The composite reliability coefficient (CR = 0.768) exceeded the recommended threshold of 0.70, indicating satisfactory internal consistency among the indicators. Additionally, the AVE value (0.539) exceeded the 0.50 benchmark, confirming adequate convergent validity. In sum, the WEMWBS demonstrated sound psychometric adequacy, indicating it is a good measure of mental health in this sample (see Table 1).

Table 1 presents the confirmatory factor analysis (CFA) fit indices, composite reliability (CR), and average variance extracted (AVE) for the study constructs in the present study. All constructs demonstrated acceptable model fit, with χ^2^/df values ranging from 1.44 to 1.92, RMSEA values between 0.041 and 0.055, and SRMR values between 0.032 and 0.048. CFI values ranged from 0.92 to 0.97, while TLI values ranged from 0.90 to 0.95, indicating satisfactory measurement-model fit (Hair et al., 2019). Regarding internal consistency, composite reliability coefficients ranged from 0.712 to 0.848, exceeding the recommended threshold of 0.70, whereas AVE values ranged from 0.532 to 0.610, supporting adequate convergent validity (Fornell and Larcker, 1981).

Item-level CFA results for the PAJFS indicated that all five items demonstrated acceptable standardized factor loadings, ranging from 0.61 to 0.82. Specifically, PAJF1 (I feel that my work utilizes my full abilities) loaded at *λ* = 0.82, PAJF2 (I feel competent and fully able to handle my job) at λ = 0.78, PAJF3 (My job gives me a chance to do things I feel I do best) at λ = 0.74, PAJF4 (I feel that my job and I are well matched) at λ = 0.61, and PAJF5 (I feel I have adequate preparation for the job I now hold) at λ = 0.68. All factor loadings exceeded the recommended minimum threshold of 0.50, indicating satisfactory indicator reliability and providing no evidence of poorly performing items. The PAJFS demonstrated good internal consistency, with a composite reliability (CR) of 0.848, exceeding the recommended criterion of 0.70. The average variance extracted (AVE = 0.532) also exceeded the recommended threshold of 0.50, supporting adequate convergent validity (Fornell and Larcker, 1981). In addition, the measurement model demonstrated acceptable fit to the data (χ^2^/df = 1.73, RMSEA = 0.050, SRMR = 0.041, CFI = 0.92, TLI = 0.90), providing further evidence for the adequacy of the measurement structure. Taken together, the item-level factor loadings, composite reliability, average variance extracted, and model fit indices support the reliability and validity of the PAJFS. Consequently, all five items were retained for the subsequent structural equation modelling analyses. All remaining constructs similarly demonstrated CR values of at least 0.70 and AVE values of at least 0.50, indicating satisfactory reliability and convergent validity across the measurement model.

### Data analysis

Preliminary data screening resulted in the removal of 28 responses due to incomplete data or response patterns indicative of inattentiveness, yielding a final analytic sample of 412 participants. Descriptive statistics and Pearson product–moment correlations were computed for all study variables as part of the preliminary analyses. All statistical analyses were executed using a two-stage computational framework. To establish the psychometric properties of the measurement scales, validity and reliability analyses, including Confirmatory Factor Analysis.

(CFA) and composite reliability estimations, were conducted using R (Version 4.5.0; R Core Team, 2026). Subsequently, the study hypotheses were formally evaluated via regression based path modeling using IBM SPSS Statistics (Version 24.0), utilizing the PROCESS macro (Model 4) to test for direct and indirect effects. Age, gender and educational qualifications, religion and marital status were included as covariates. Indirect effects were regarded as statistically significant when the 95% bootstrapped confidence intervals excluded zero. Given the reliance on self-report data, several procedural safeguards were implemented to mitigate potential common method variance (CMV). Participants were assured of anonymity and response confidentiality to reduce social desirability concerns. Measures were presented in distinct sections to psychologically separate constructs, item ordering within sections was randomized to minimize response set tendencies, and select items were reverse-scored to encourage careful responding. Harman’s single-factor test was subsequently conducted as a statistical check for CMV. Five factors yielded eigenvalues exceeding 1.0, with the first factor accounting for 38.7% of total variance — below the conventional 50% threshold — suggesting that common method bias did not pose a substantive threat to the integrity of the findings (Podsakoff et al., 2003). Although Harman’s single-factor test serves as a constrained diagnostic tool for identifying common method variance, it may fail to detect nuanced or intricate biases within self-reported data. Consequently, common method variance cannot be entirely dismissed, necessitating a cautious interpretation of the observed relationships, as these associations may be inflated (Podsakoff et al., 2003).

## Results

Table 2 presents the bivariate correlations among the study variables. Mental health was significantly and negatively correlated with mindfulness (*r* = −0.39, *p* < 0.001), indicating that higher trait mindfulness was associated with lower mental health scores in this sample. By contrast, mental health showed significant positive correlations with EI (*r* = 0.30, *p* < 0.001) and person–job fit (*r* = 0.14, *p* < 0.05). Workplace stress was not significantly associated with mental health (*r* = −0.02, *p* > 0.05). Notably, mindfulness showed a significant negative correlation with EI (*r* = −0.31, *p* < 0.001), suggesting that higher trait mindfulness was associated with lower emotional intelligence in this sample. Workplace stress showed a weak but significant positive correlation with EI (*r* = 0.14, *p* < 0.05). All remaining correlations among the focal study variables were non-significant. Demographic variables (age, gender, ethnicity, religion, marital status, education, and bank affiliation) did not yield significant correlations with mental health.

**Table 2:** Correlations between study variables (*N* = 412).

**Variable**	**Mean SD**		**1**	**2**	3	4	**5**	**6**	**7**	**8**	**9**	**10**	**11**	**12**
1. Age	35.48	7.29	–											
2. Gender	–	–	–0.13	–										
3. Ethnicity	–	–	**–0.14** ^*^	**0.24** ^***^	–									
4. Religion	–	–	0.01	**–0.13** ^*^	0.02	–								
5. Marital status	–	–	**0.18** ^**^	–0.10	0.07	0.23***	–							
6. Educational qualification	–	–	–0.09	0.05	0.02	0.04	0.39***	–						
7. Bank	–	–	**–0.16**	–0.01	0.05	–0.04	0.03	0.35***	–					
8. Mindfulness	34.54	7.02	**0.16** ^*^	0.05	–0.03	0.11	0.23***	0.09	–0.06	–				
9. Employee job fit	13.94	3.00	0.04	0.08	0.01	–0.01	0.09	0.01	0.03	0.04	–			
10. Workplace stress	14.98	3.50	0.04	0.08	–0.02	–0.02	**–0.16** ^*^	–0.01	–0.01	0.09	0.10	–		
11. Emotional intelligence	35.17	7.81	–0.10	0.06	0.09	0.10	**–0.15** ^*^	–0.03	–0.01	**–0.31** ^***^	0.10	**0.14** ^*^	–	
12. Mental health	48.42	10.87	–0.10	0.03	0.08	–0.06	–0.01	0.06	0.11	**–0.39** ^***^	**0.14** ^*^	–0.02	**0.30** ^***^	–

^***^ < 0.001; ^**^ < 0.01; ^*^p < 0.05 (Gender: Male = 0, Female = 1); Bold indicates significant correlations.

**Figure 2:**
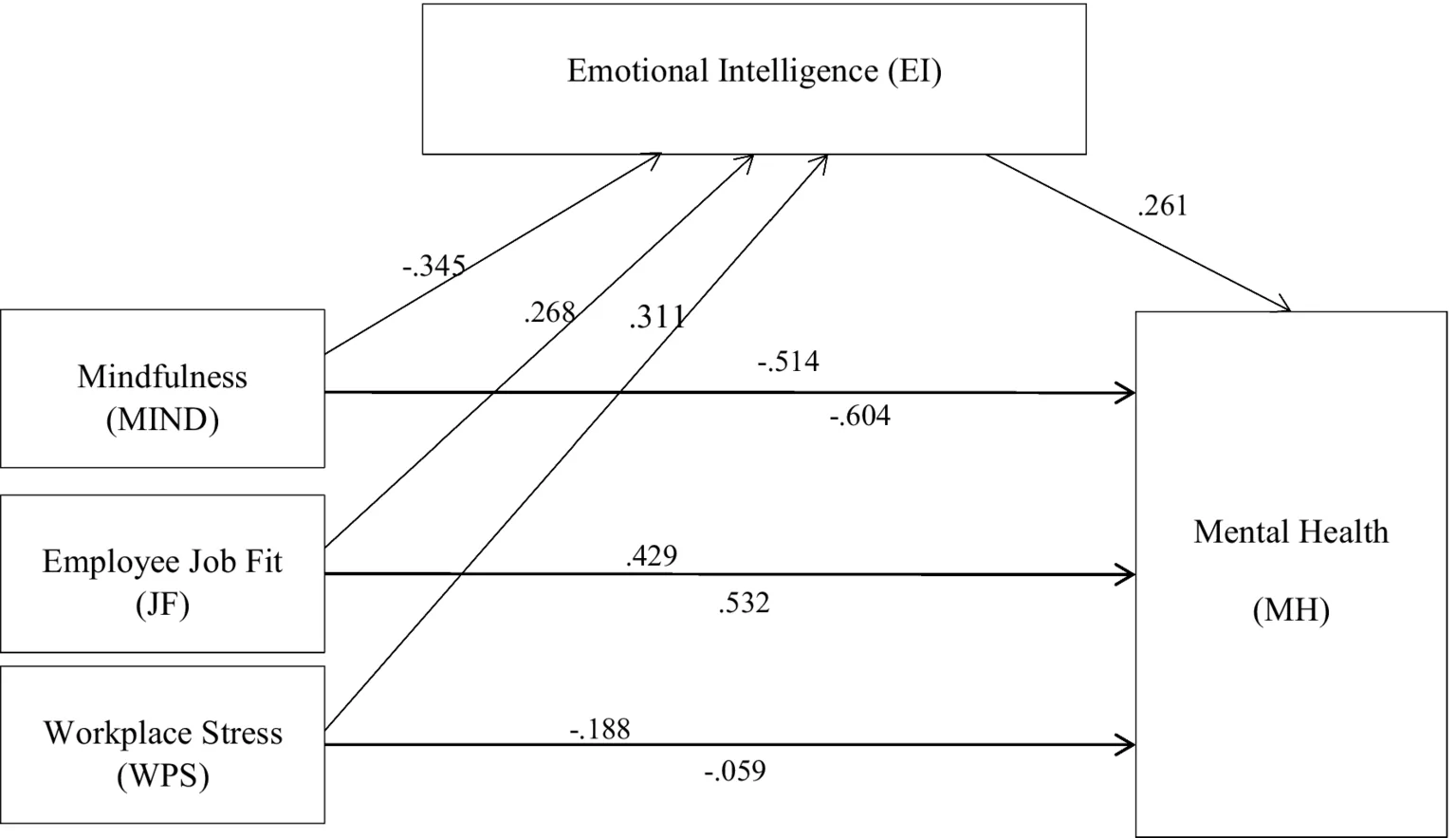
Statistical model for the mediating role of emotional intelligence in the relationships of workplace stress, mindfulness, and employee job–fit with mental health.

Table 3 and Figure 2 presents the mediation analysis results. Total effects (path *c*) represent the influence of each independent variable on mental health in the absence of EI as a mediator; direct effects (path *c*′) represent the influence of each predictor on mental health with EI held constant; and indirect effects (path *ab*) capture the extent to which EI contributed to each predictor–mental health relationship.

**Table 3:** Results for the mediating role of emotional intelligence in the relationship between workplace stress, mindfulness, employees’ job fit, and mental health.

Prediction	Effect (Path)	B	SE	T	BCa 95% CI [LLCI, ULCI]	Remark
IV1: Workplace stress (WPS)						
WPS → EI	(a_3_)	0.311	0.144	**2.16** ^*^	[0.028, 0.594]	No mediation
R^2^ M,X		0.031				
EI → MH	(b_3_)	0.414	0.088	**4.72** ^***^	[0.241, 0.587]	
WPS → MH	Direct Effect (c′_3_)	–0.188	0.195	–0.96	[–0.573, 0.197]	
R^2^ Y,MX		0.097				
WPS → EI → MH Indirect Effect (ab_3_)	0.129	0.076	[–0.011, 0.285]			
WPS → MH	Total Effect (c_3_)	–0.059	0.202	–0.29	[–0.457, 0.339]	
R^2^ Y,X		0.011				
IV2: Mindfulness (MIND)						
MINDF → EI	(a_1_)	–0.345	0.070	**–4.97** ^***^	[–0.482, –0.208]	Mediated
R^2^ M,X		0.106				
EI → MH	(b_1_)	0.261	0.086	**3.03** ^**^	[0.091, 0.432]	
MINDF → MH	Direct Effect (c′_1_)	–0.514	0.097	**-5.30** ^***^	[–0.705, –0.323]	
R^2^ Y,MX		0.190				
MINDF → EI → MH	Indirect Effect (ab_1_)	–0.090	0.038		[–0.177, –0.030]	
MINDF → MH	Total Effect (c_1_)	–0.604	0.094	**–6.44** ^***^	[–0.789, –0.419]	
R^2^ Y,X		0.159				
IV3: Job fit (JobFit)						
JobFit → EI	(a_2_)	0.268	0.168	1.59	[–0.064, 0.600]	No mediation
R^2^ M,X		0.022				
EI → MH	(b_2_)	0.385	0.087	**4.44** ^***^	[0.214, 0.556]	
JobFit → MH	Direct effect (c′_2_)	0.429	0.226	1.90	[–0.015, 0.873]	
R^2^ Y,MX		0.107				
JobFit → EI → MH indirect effect (ab_2_)	0.103	0.080			[–0.022, 0.295]	
JobFit → MH	Total effect (c_2_)	0.532	0.233	**2.28** ^*^	[0.073, 0.991]	
R^2^ Y,X		0.032				

**p < 0.001; *p < 0.01; *p* < 0.05. B = unstandardized coefficient; SE = standard error; BCa CI = bias-corrected and accelerated confidence interval; LLCI = lower-level confidence interval; ULCI = upper-level confidence interval; MINDF = Mindfulness; JobFit = Employees’ Job Fit; WPS = Workplace Stress; MH = Mental Health; EI = Emotional Intelligence. Path a = effect of X on M; Path b = effect of M on Y (controlling for X); Path c′ = direct effect of X on Y (controlling for M); Path c = total effect of X on Y; ab = indirect (mediated) effect of X on Y through M; R^2^ M, X = variance in mediator explained by X; R^2^ Y, MX = variance in outcome explained by X and M; R^2^ Y, X = variance in outcome explained by X only. Bold values represent significance.

Regarding the workplace stress pathway, the direct effect of workplace stress on EI was significant and positive (*B* = 0.311, *p* < 0.05, 95% CI [0.028, 0.594]), contrary to the direction predicted by H4, which proposed a negative relationship. EI significantly associated with mental health (*B* = 0.414, *p* < 0.001, 95% CI [0.241, 0.587]). However, the direct effect of workplace stress on mental health was non-significant (*B* = −0.188, *p* > 0.05, 95% CI [−0.573, 0.197]), the indirect effect through EI was also non-significant (effect = 0.129, 95% CI [−0.011, 0.285]), and the total effect of workplace stress on mental health failed to reach significance (*B* = −0.059, *p* > 0.05, 95% CI [−0.457, 0.339]). Collectively, H1, H4, and H8 were not supported.

Regarding the mindfulness pathway, the direct effect of mindfulness on EI (path *a*) was significant and negative (*B* = −0.345, *p* < 0.001, 95% CI [−0.482, −0.208]), indicating that higher mindfulness was associated with lower EI. EI significantly and positively associated with mental health (path *b*; *B* = 0.261, *p* < 0.01, 95% CI [0.091, 0.432]), such that employees with higher EI reported better mental health. The direct effect of mindfulness on mental health (path *c*′) was also significant and negative (*B* = −0.514, *p* < 0.001, 95% CI [−0.705, −0.323]). The indirect effect of mindfulness on mental health via EI (path *ab*) was significant (effect = −0.090, 95% CI [−0.177, −0.030]), as the confidence interval excluded zero, indicating partial mediation. Specifically, EI partially reduced the negative direct effect of mindfulness on mental health, reducing the effect from −0.514 to −0.090. The total effect of mindfulness on mental health remained significant and negative (*B* = −0.604, *p* < 0.001, 95% CI [−0.789, −0.419]), supporting H9 (EI mediates the mindfulness–mental health relationship).

Regarding the person–job fit pathway, the direct effect of PJF on EI was positive but non-significant (*B* = 0.268, *p* > 0.05, 95% CI [−0.064, 0.600]), failing to support H6. EI nonetheless significantly associated with mental health in this model (*B* = 0.385, *p* < 0.001, 95% CI [0.214, 0.556]). The direct effect of PJF on mental health was marginally non-significant (*B* = 0.429, *p* > 0.05, 95% CI [−0.015, 0.873]), while the total effect reached significance (*B* = 0.532, *p* < 0.05, 95% CI [0.073, 0.991]), indicating that PJF exerts an overall positive effect on mental health when indirect pathways are considered, thus partially supporting H3. The indirect effect of PJF on mental health through EI was non-significant (effect = 0.103, 95% CI [−0.022, 0.295]), as the confidence interval included zero, indicating that EI did not mediate this relationship. H10 was therefore rejected.

Overall, EI served as a significant partial mediator only in the mindfulness–mental health pathway (H9 supported), while mediation was not evident for the PJF–mental health (H10 rejected) or workplace stress–mental health (H8 rejected) pathways. EI consistently and positively associated with mental health across all three models, supporting H7.

## Discussion

The present study examined the roles of workplace stress, mindfulness, and person–job fit in association with employee mental health among bank employees in Nigeria, with EI investigated as a mediator. The findings yielded a nuanced pattern of results that both converge with and diverge from existing theoretical expectations, offering important contributions to occupational health psychology. The first hypothesis, which proposed that workplace stress would negatively associate with employee mental health, was not supported. Both the direct and total effects of workplace stress on mental health were non-significant. This finding is somewhat surprising given the extensive body of literature documenting the deleterious effects of occupational stress on mental health and psychological well-being (Nixon et al., 2011; Stansfeld and Candy, 2006). One possible explanation is that the sample—drawn from bank employees in Nigeria may represent a relatively resilient occupational group habituated to high-pressure environments, thereby reducing any direct stress–health association. This interpretation is consistent with Conservation of Resources (COR) theory (Hobfoll, 1989), which suggests that individuals with robust personal resources may buffer the adverse effects of stress. Moreover, the absence of a significant association may suggest the existence of unmeasured buffering boundary conditions that mitigate the impact of stress on mental health. Within the Job Demands–resources (JD-R) framework, resources such as resilience have been shown to enhance employees’ capacity to maintain psychological functioning under high job demands, thereby reducing adverse stress outcomes (Bakker and Demerouti, 2017; Fletcher and Sarkar, 2013). Similarly, perceived social support from supervisors and colleagues consistently emerges as a protective factor that alleviates the negative psychological effects of occupational stress in high-stress service environments, including the banking sector (Cohen and Wills, 1985; Rhoades and Eisenberger, 2002). Recent occupational health studies in the banking sector have further corroborated this buffering role (Iype and Aithal, 2024; Yıldırım and Darıcan, 2024). Consequently, these resources may suppress the direct manifestation of stress effects on mental health, resulting in a non-significant overall association. These findings suggest that the relationship between stress and mental health is likely contingent rather than linear (Stansfeld and Candy, 2006) and may be significantly influenced by individual and contextual factors. Therefore, future research should incorporate key psychological and organizational resources within a moderated JD-R framework to more accurately capture these conditional effects in banking populations.

Contrary to the second hypothesis, mindfulness demonstrated a significant and negative association with mental health, indicating that higher mindfulness was associated with poorer self-reported mental health outcomes. This unexpected finding challenges the predominant narrative that mindfulness uniformly benefits mental health and psychological well-being (Khoury et al., 2015; Lomas et al., 2017). However, a growing body of critical scholarship has begun to question the universality of mindfulness benefits, particularly in demanding organizational contexts (Van Dam et al., 2018). It is plausible that in a high-demand banking environment, elevated mindfulness such as trait mindfulness may paradoxically heighten employees’ awareness of work-related distress and burnout—a phenomenon described as the “dark night of the soul” in contemplative literature (Lindahl et al., 2017). Trait mindfulness holds particular significance within the Nigerian banking sector as it constitutes a stable personal resource that facilitates employees’ ability to regulate attention, manage emotions, and effectively address ongoing occupational demands. This interpretation is supported by evidence that heightened non-reactive awareness can amplify the perceived salience of negative emotional experiences before adaptive regulation is engaged (Brown et al., 2007). Moreover, the unexpected results can be theoretically interpreted through the Job Demands– Resources (JD-R) model (Bakker and Demerouti, 2007, 2017) and the emerging evidence that trait mindfulness functions as a stable psychological resource in the workplace. For example, in high-demand, underexplored occupational environments, such as the banking sector in Nigeria, increased mindfulness may enhance awareness of ongoing stressors and emotional strain without providing sufficient coping resources to deal with them. In this context, heightened attentional clarity may initially increase the prominence of job-related demands, resulting in more negative evaluations of mental health outcomes than immediate psychological benefits. This interpretation is consistent with recent empirical evidence suggesting that mindfulness is not always a uniformly beneficial resource and may, under certain conditions, as observed in the present study, be associated with heightened emotional awareness of distress and reduced mental health, particularly in high-stress or resource constrained environments (e.g., Good et al., 2016; Workman and Beer, 2018). Additionally, from a Conservation of Resources (COR) perspective (Hobfoll, 1989), individuals with depleted psychological resources may experience mindfulness as an increased awareness of resource loss rather than effective regulation, which can temporarily coincide with lower perceived emotional intelligence as individuals become more aware of their emotional difficulties. In contrast to state mindfulness, which is transient and context-dependent, or mindfulness interventions that require continuous organizational support and practice, trait mindfulness offers a persistent capacity for self-regulation in challenging work environments (Mesmer-Magnus et al., 2017). This distinction is particularly relevant in Nigeria’s banking industry, where high-performance pressure and limited formal well-being resources may necessitate greater reliance on individual psychological resources. These findings suggest that trait mindfulness functions as a stable psychological resource in occupational settings, such as banking, and may be moderated by prevailing job demands and available coping resources, aligning with recent calls to reconsider the assumption of uniformly positive mindfulness outcomes in organizational settings. Therefore, rather than contradicting the existing literature, the results underscore the important boundary conditions under which mindfulness may operate differently within the JD-R framework, warranting further investigation of the conditions under which mindfulness promotes or undermines mental health in high-demand work environments such as the banking sector.

The third hypothesis, which predicted that PJF would positively associate with employee mental health, received marginal support. Although the direct effect of PJF on mental health narrowly missed conventional significance thresholds, the significant total effect indicates that PJF exerts a meaningful overall influence on mental health when indirect pathways are considered. These findings are broadly consistent with person–environment fit theory (Kristof-Brown et al., 2005) and with recent evidence from healthcare and service sector contexts demonstrating that congruence between individual competencies and job demands promotes psychological well-being and reduces burnout (Zeng and Hu, 2024; Weng et al., 2025). The borderline direct effect may reflect the complex, contingent nature of fit perceptions in high- pressure organizational settings (Brandstätter et al., 2016), where PJF alone may be insufficient to overcome structural stressors without additional personal or organizational resources.

The fourth hypothesis, which proposed that workplace stress would negatively associate with EI was not supported; rather, a significant positive relationship was observed. Employees who reported higher workplace stress also reported higher EI. This counterintuitive finding invites several theoretical interpretations derived from the Job Demands–Resources.

(JD–R) model and the challenge–hindrance stressor framework. These theoretical perspectives.

suggest that job demands are not inherently harmful; rather, when perceived as challenge stressors, they can facilitate learning, adaptation, and skill development (Bakker and Demerouti, 2017; LePine et al., 2005). For instance, in the Nigerian banking sector, employees frequently encounter high levels of customer interaction, performance targets, and emotionally charged service situations. These conditions may act as developmental stressors necessitating ongoing emotion regulation, empathy, and interpersonal competence. Over time, consistent exposure to these demands may enhance employees’ emotional intelligence instead of diminishing it. Furthermore, perspectives on stress-related growth indicate that manageable stress can improve coping abilities and emotional skills (Folkman and Moskowitz, 2000). Therefore, in this context, workplace stress may function as a mechanism for experiential learning and occupational socialization, enabling employees to progressively acquire and refine the emotional competencies essential for effective service delivery.

The fifth hypothesis, which posited that mindfulness would positively associate with EI, was also not supported. This finding diverges from theoretical frameworks that commonly posit mindfulness as a facilitator of emotional awareness and regulation (Jiménez-Picón et al., 2021; Schutte et al., 2021). In the context of Nigerian banking, this phenomenon can be interpreted through the Job Demands–Resources model, which suggests that high job demands and limited resources influence the deployment of personal capacities (Bakker and Demerouti, 2017). The banking sector in Nigeria is marked by significant performance pressure, emotional labor, and customer-facing demands, potentially leading employees to adopt mindfulness as a strategy for cognitive efficiency rather than emotional enhancement. Under these circumstances, trait mindfulness may serve as a mechanism for attentional detachment, thereby reducing emotional elaboration to conserve cognitive resources for task performance. As a result, employees with high mindfulness may engage in less explicit emotional processing, which could account for the observed decrease in EI. Furthermore, EI in the banking sector (e.g., Nigeria) may be more influenced by organizational norms and role expectations than by individual dispositional factors. Therefore, in this environment, mindfulness may represent adaptive self-regulation in response to chronic stress rather than an indication of enhanced emotional competence.

The sixth hypothesis, which proposed that PJF would positively associate with EI was not supported, as the path from PJF to EI was non-significant suggesting that employees who fit their roles well may have more cognitive and motivational resources available for emotional processing. While the positive trend is theoretically coherent, aligning with the person– environment fit framework which posits that an improved fit enhances cognitive and motivational resources for emotional processing (Kristof-Brown et al., 2005; Edwards, 2006). However, the lack of a significant relationship between P-J fit and emotional intelligence may be attributed to the limited variability in fit perceptions within a relatively homogeneous occupational sample, such as the Nigerian banking sector. Furthermore, ceiling effects in emotional intelligence may be present in this banking context due to selection and socialization processes inherent in emotionally demanding service roles (Joseph and Newman, 2010; McShane and Von Glinow, 2018). Future research with more heterogeneous samples and objective fit indices should revisit this pathway.

Hypothesis seven was robustly supported: EI positively associate with employee mental health across all three mediation models. This finding is consistent with the ability model of EI (Mayer et al., 2000) and mixed model perspectives (Bar-On, 2006), both of which emphasize EI as a functional competency enabling adaptive coping, positive interpersonal functioning, and self-regulation resources that directly sustain mental health. The consistency of this effect across all models strengthens calls for the integration of EI development into employee wellbeing interventions (Galea et al., 2025; Li et al., 2023; Slaski and Cartwright, 2002). Recent evidence from healthcare and high-demand service settings similarly demonstrates that EI functions as a robust protective factor against burnout and psychological distress (Han et al., 2025; He et al., 2024).

Hypothesis eight was rejected: EI did not serve as a significant mediator in the workplace stress–mental health pathway. Given that workplace stress showed a positive rather than negative relationship with EI, the precondition for mediation via the hypothesized mechanism was not met. This absence of mediation is statistically coherent with the overall pattern of results and aligns with more recent calls to contextualize mediation models within specific occupational and cultural settings (Brunetto et al., 2012; Joseph and Newman, 2010). The finding suggests that in the Nigerian banking context, the relationship between workplace stress and mental health may operate through alternative mechanisms—such as social support, avoidance coping, or structural job demands–resources processes—that require further empirical investigation.

Hypothesis nine was supported: EI significantly and partially mediated the mindfulness–mental health relationship. Specifically, EI attenuated the otherwise adverse direct association between mindfulness and mental health. This is a theoretically important finding. It suggests that although heightened mindfulness such as trait in this occupational context may initially amplify awareness of negative emotional states—consistent with the decentering and heightened sensitivity hypotheses (Fresco et al., 2007; Van Dam et al., 2018)—the EI competencies associated with mindful employees partially mediated this heightened awareness into adaptive emotional regulation, yielding better mental health outcomes than would be expected from the direct effect alone. This novel finding contributes to the occupational health psychology literature by specifying EI as a critical pathway through which the effects of mindfulness (e.g., trait mindfulness) on mental health can be partially or fully mediated in high-demand work environments, and supports theoretical integration of mindfulness and EI frameworks (Brown et al., 2007; Long et al., 2025; Schutte and Malouff, 2011).

Finally, hypothesis ten was rejected: EI did not mediate the PJF–mental health relationship. Given that PJF did not significantly predict EI, the absence of mediation is consistent with contemporary bootstrapping frameworks for mediation inference (Preacher and Hayes, 2008; Hayes, 2018). The significant total effect of PJF on mental health nonetheless suggests that PJF exerts a meaningful influence through pathways other than EI—possibly through direct fulfilment of basic psychological needs for competence and autonomy (Ryan and Deci, 2000), enhanced work motivation, or reduced role ambiguity. Future research should examine whether other personal resources (e.g., self-efficacy, psychological capital) mediate the PJF–mental health relationship in banking context.

### Implications of the study

The findings of the present study carry several important implications for organizational practice, particularly the banking sector. First, the robust and consistent positive relationship between EI and mental health across all models underscores the strategic value of EI within organizations, particularly in the banking sector in Nigeria. Human resource departments and occupational health practitioners should consider embedding structured EI training, encompassing emotion appraisal, self-regulation, and utilization competencies into professional development curricula for employees, as such interventions have demonstrated efficacy in improving well-being and reducing burnout in analogous high-demand settings (Galea et al., 2025; Slaski and Cartwright, 2002).

Second, the finding that EI mediates the mindfulness–mental health pathway in a suppressive direction highlights a critical boundary condition for mindfulness-based organizational interventions. Rather than implementing generic mindfulness programs.

Organizations should consider integrating mindfulness training with explicit EI development components, so that the heightened emotional awareness associated with mindfulness (e.g., trait mindfulness) is channeled through adaptive regulatory strategies rather than amplifying distress.

Third, the significant total effect of PJF on mental health highlights the importance of systematic person–job fit alignment in human resource management, particularly in banking sector. Organizations should invest in rigorous recruitment, on-boarding, and role-design processes that ensure congruence between employees’ skills, abilities, and values and the demands and characteristics of their roles. Regular job crafting opportunities and role review processes may help maintain and improve perceived PJF over time, thereby sustaining employee mental health (Kristof-Brown et al., 2005; Zeng and Hu, 2024).

Finally, the non-significant direct and total effects of workplace stress on mental health in this sample should not be interpreted as an indication that stress management is unimportant. Rather, it suggest that structural and systemic interventions—including workload management, adequate staffing, clear performance expectations, and institutional mental health support, remain essential to creating work environments, particularly in the banking sector that do not deplete employees’ psychological resources.

In summary, the findings challenge simplistic assumptions about the universally protective roles of mindfulness and the universally harmful effects of workplace stress, and instead highlight the importance of occupational context and personal resources in shaping mental health outcomes. The consistent predictive utility of emotional intelligence across models underscores its status as a robust wellbeing resource in work environments, even when its mediating role is context-dependent. Practically, the findings suggest that organizations, such as the Nigerian banking sector seeking to promote employee mental health would benefit from investing in emotional intelligence training and development programs. In addition, efforts to optimize person–job fit—through improved recruitment, role design, and career development—may yield downstream mental health benefits that do not rely on emotional intelligence as an intermediary.

### Limitations and future directions

This study has several limitations. First, the reliance on self-report measures introduces the potential for common method variance (Podsakoff et al., 2003). However, this concern was mitigated through the use of a two-wave design, which temporally separated the measurement of study variables and strengthened the temporal ordering of pathways in the mediation models. Future research should incorporate multi-source assessments of emotional intelligence, including performance-based measures of emotional intelligence. Second, the sample was confined to bank employees within a specific geographical context such as South-east Nigeria, which may limit the extrapolation and generalizability of the findings; thus, cross-cultural and cross-industry replications are encouraged. Finally, the unexpected negative association of mindfulness necessitates replication and further theoretical elaboration in various occupational and cultural contexts. Future research should investigate whether the approach to measuring mindfulness (trait versus practice frequency) and the extent of exposure influence these effects.

## Conclusion

This study examined the mediating role of EI in the relationships of workplace stress, mindfulness and person–job fit on mental health among bank employees in Nigeria. The central and most theoretically significant finding is that EI significantly and partially mediated the relationship between mindfulness and mental health, in a direction that partially reduced the otherwise negative direct effect of mindfulness on mental health. This suppressive mediation pattern indicates that mindfulness, particularly trait dimension of mindfulness in this high demand occupational context amplified awareness of negative affect and diminished EI, yet the EI competencies associated with mindful employees partially mediated this heightened awareness into adaptive emotional regulation, thereby buffering its adverse impact on mental health. This finding challenges the assumption that mindfulness is uniformly beneficial and identifies EI capacity as a critical intervening condition for its effectiveness in organizational contexts (e.g., banking). Person–job fit emerged as a significant overall predictor of mental health, with its total effect reaching significance despite a non-significant direct effect, suggesting that PJF influences mental health through pathways other than EI—likely including direct fulfilment of psychological needs for competence, autonomy, and role clarity. Workplace stress showed neither a significant direct nor total effect on mental health, and EI did not mediate either the PJF–mental health or workplace stress–mental health pathways. Across all models, EI consistently and positively associated with mental health, underscoring its role as a robust personal resource for mental health in organizations (e.g., banking sector in Nigeria). These findings advance theoretical understanding by demonstrating, within an integrated JD-R and Emotion Regulation Theory framework that EI serves as a proximal, process-oriented mechanism that can partially mediate the influence of distal personal resources such as mindfulness on mental health outcomes. The findings also demonstrate the importance of contextualizing psychological resource frameworks within specific occupational and cultural settings, as relationships that have been theorized and observed in Western samples may manifest differently in Sub-Saharan African work environments as witnessed in Nigerian banking sector. Thus, the findings advocate for integrated, dual-component wellness interventions that cultivate both mindfulness and EI, rather than implementing them in isolation alongside systemic human resource policies that prioritize person–job alignment, sustainable workload management and EI development as core components of occupational health strategy, especially in an industry as psychologically demanding as banking sector in Nigeria.

Hence, understanding and addressing this mechanisms that protect or erode mental health is not merely a welfare concern, but a strategic organizational necessity, particularly in a high pressure work environment such as the banking sector.

## Data Availability

The raw data supporting the conclusions of this article will be made available by the authors, without undue reservation.
